# Casorati Inequalities for Statistical Submanifolds in Kenmotsu Statistical Manifolds of Constant *ϕ*-Sectional Curvature with Semi-Symmetric Metric Connection

**DOI:** 10.3390/e24060800

**Published:** 2022-06-08

**Authors:** Simona Decu, Gabriel-Eduard Vîlcu

**Affiliations:** 1 Department of Applied Mathematics, Bucharest University of Economic Studies, 6 Piaţa Romană, 010374 Bucharest, Romania; 2 Romanian Academy, Center of Mountain Economy (CE-MONT), “Costin C. Kiriţescu” National Institute of Economic Research, 13 Calea 13 Septembrie, 030508 Bucharest, Romania; 3 Department of Mathematics and Informatics, Faculty of Applied Sciences, University Politehnica of Bucharest, Splaiul Independenţei 313, 060042 Bucharest, Romania; gvilcu@upg-ploiesti.ro; 4 Romanian Academy, “Gheorghe Mihoc-Caius Iacob” Institute of Mathematical Statistics and Applied Mathematics, 050711 Bucharest, Romania; 5 Department of Cybernetics, Economic Informatics, Finance and Accountancy, Petroleum-Gas University of Ploieşti, Bd. Bucureşti 39, 100680 Ploieşti, Romania

**Keywords:** statistical manifold, semi-symmetric metric connection, Casorati curvature, Kenmotsu statistical manifold

## Abstract

In this paper, we prove some inequalities between intrinsic and extrinsic curvature invariants, namely the normalized δ-Casorati curvatures and the scalar curvature of statistical submanifolds in Kenmotsu statistical manifolds of constant ϕ-sectional curvature that are endowed with semi-symmetric metric connection. Furthermore, we investigate the equality cases of these inequalities. We also describe an illustrative example.

## 1. Introduction

The study of simple relationships between the main intrinsic and extrinsic invariants of submanifolds is a fundamental problem in submanifold theory [1]. Recent research shows a growing trend in approaching this fascinating problem through an approach that proves some types of geometric inequalities (see, e.g., [2,3,4,5,6,7,8,9,10]).

The interest in such inequalities goes back in 1993, when B.-Y. Chen introduced the intrinsic *δ-invariants*, now called *Chen invariants*, satisfying optimal inequalities for submanifolds in real space forms [11]. Later, the notion of normalized *δ-Casorati curvatures* (extrinsic invariants) was defined in [12,13], giving rise to new inequalities. Unlike the Gauss and mean curvature, F. Casorati in 1890 proposed to measure the curvature of a surface at a point according to common intuition of curvature [14]. Currently, this measure is named the *Casorati curvature*, defined by $C=\frac{k_{1}^{2}+k_{2}^{2}}{2}$, where $k_{1}$ and $k_{2}$ are the principal curvatures of the surface in $E^{3}$. L. Verstraelen geometrically modeled the perception as the Casorati curvature of sensation in the context of early human vision [15]. The Casorati curvature is also assessed as a natural measure or a measure of the normal deviations from planarity in some models of computer vision [16,17]. In mechanics and modern computer science, the Casorati curvature has become known as *bending energy* [17].

The topic of δ-Casorati curvatures will appeal to more geometers focused on finding new solutions of the above problem. In this respect, some recent developments are devoted to inequalities on various submanifolds of a *statistical manifold*, notion defined by Amari [18] in 1985 in the realm of information geometry [3,4,5,6,7,8,9,10]. In this setting, the Fisher information metric is one of the most important metrics that can be considered on statistical models [19]. Actually, it is known that modulo rescaling is the only Riemannian metric invariant under sufficient statistics and it is seen as an infinitesimal form of the relative entropy [20]. In particular, Fisher information metrics play a key role in the multiple linear regressions by maximizing the likelihood [21]. Statistical manifolds are also applied in fields such as physics, machine learning, statistics, etc. There is a natural relationship between statistical manifolds and entropy. For example, P. Pessoa et al. studied the entropic dynamics on the statistical manifolds of Gibbs distributions in [22]. Since each point of the space is a probability distribution, a statistical manifold has a profound effect on the dynamics.

Initiated by K. Kenmotsu in 1972 [23] as a branch of contact geometry, *Kenmotsu geometry* has generated a wide range of applications in physics (thermodynamics, classical mechanics, geometrical optics, geometric quantization, classical mechanics) and control theory [24]. The *Kenmotsu statistical manifold*, defined by H. Furuhata in [25], is obtained locally as a warped product between a holomorphic statistical manifold and a real line. In [8], the authors established some Casorati inequalities for Kenmotsu statistical manifolds of constant ϕ-sectional curvature.

The concept of *semi-symmetric metric connection* on a Riemannian manifold was introduced by H.A. Hayden in [26]. Later, interesting properties of a Riemannian manifold with semi-symmetric metric connection were obtained by K. Yano in [27] and T. Imai in [28]. In addition, T. Imai investigated hypersurfaces of a Riemannian manifold with semi-symmetric metric connection [29]. Z. Nakao generalized Imai’s approach of hypersurfaces by studying submanifolds of a Riemannian manifold with semi-symmetric metric connection [30]. The geometric inequalities on submanifolds in various manifolds with semi-symmetric metric connection have been extensively proven (see, e.g., [31,32,33,34,35,36,37]). However, only a few results are dedicated to the ambient of statistical manifolds endowed with semi-symmetric metric connection. S. Kazan and A. Kazan obtained some geometric properties of Sasakian statistical manifolds with a semi-symmetric metric connection [38]. Furthermore, M.B.K. Balgeshir and S. Salahvarzi studied new curvature properties and equations of statistical manifolds with a semi-symmetric metric connection as well as their submanifolds [39].

In this article, we establish some basic inequalities between the normalized δ-Casorati curvatures (that are known to be extrinsic invariants) and the scalar curvature (a fundamental intrinsic invariant) of statistical submanifolds in Kenmotsu statistical manifolds having a constant ϕ-sectional curvature, which are endowed with semi-symmetric metric connection. Moreover, we investigated the equality cases of such inequalities. A nontrivial example is also constructed in the last part of the article.

## 2. Preliminaries

Let ($\bar{M}$, *g*) be a Riemannian manifold, with *g* a Riemannian metric on $\bar{M}$ and $\bar{\nabla }$ an affine connection on $\bar{M}$. A triplet ($\bar{M}$, *g*, $\bar{\nabla }$) is called a *statistical manifold* if the torsion tensor field of $\bar{\nabla }$ vanishes and $\bar{\nabla }g$ is symmetric [40]. With other words, the pair ($\bar{\nabla }$, *g*) is a statistical structure on $\bar{M}$. Let ${\bar{\nabla }}^{*}$ be an affine connection of $\bar{M}$ defined by

$$X\;\bar{g}\left(Y,Z\right)=\bar{g}\left({\bar{\nabla }}_{X}Y,Z\right)+\bar{g}\left(Y,{\bar{\nabla }}_{X}^{*}Z\right),$$

for any *X*, *Y*, *Z*$\in \Gamma (T\bar{M})$, where $\Gamma (T\bar{M})$ is the set of smooth tangent vector fields on $\bar{M}$.

Then ${\bar{\nabla }}^{*}$ is named the *dual connection* of $\bar{\nabla }$ with respect to *g*. Clearly, ${\left({\bar{\nabla }}^{*}\right)}^{*}=\bar{\nabla }$. Moreover, the Levi-Civita connection on $\bar{M}$ is given by ${\bar{\nabla }}^{0}=\frac{\bar{\nabla }+{\bar{\nabla }}^{*}}{2}$ [41]. If $(\bar{M}$, *g*, $\bar{\nabla })$ is a statistical manifold, then it is known that $(\bar{M}$, *g*, ${\bar{\nabla }}^{*})$ is too.

Let *M* be a submanifold of a statistical manifold $(\bar{M}$, *g*, $\bar{\nabla })$ with *g* the induced metric on *M*, and ∇ the induced connection on *M*. Then (M,g,∇) is a statistical manifold as well.

Denote by *h* and $h^{*}$ the *imbedding curvature tensor* of *M* in $\bar{M}$ with respect to $\bar{\nabla }$ and ${\bar{\nabla }}^{*}$, respectively. Then Gauss’s formulas [40] are expressed by:

$$\begin{array}{c} {\bar{\nabla }}_{X}Y=\nabla_{X}Y+h\left(X,Y\right),\\ {\bar{\nabla }}_{X}^{*}Y=\nabla_{X}^{*}Y+h^{*}\left(X,Y\right), \end{array}$$

for any X,Y∈Γ(TM).

Furthermore, denote by *R*, $\bar{R}$, $R^{*}$ and ${\bar{R}}^{*}$ the (0,4)-*curvature tensors* for the connections ∇, $\bar{\nabla }$, $\nabla^{*}$ and ${\bar{\nabla }}^{*}$, respectively. Thus, the *Gauss equations* for the connections $\bar{\nabla }$ and ${\bar{\nabla }}^{*}$, respectively, hold as follows [41]:

(1) $$\begin{array}{ccc} g(\bar{R}\left(X,Y\right)Z,W) & = & g\left(R\left(X,Y\right)Z,W\right)+g\left(h\left(X,Z\right),h^{*}\left(Y,W\right)\right)\\ & - & g(h^{*}\left(X,W\right),h\left(Y,Z\right)), \end{array}$$

and

(2) $$\begin{array}{ccc} g({\bar{R}}^{*}\left(X,Y\right)Z,W) & = & g\left(R^{*}\left(X,Y\right)Z,W\right)+g\left(h^{*}\left(X,Z\right),h\left(Y,W\right)\right)\\ & - & g(h\left(X,W\right),h^{*}\left(Y,Z\right)), \end{array}$$

for any X,Y,Z,W∈Γ(TM).

We can define now the *statistical curvature tensor field* [40] on *M* and $\bar{M}$, denoted by *S* and $\bar{S}$, respectively:

(3) $$S\left(X,Y\right)Z=\frac{1}{2}\left\{R\left(X,Y\right)Z+R^{*}\left(X,Y\right)Z\right\},$$

for any X,Y,Z,W∈Γ(TM), and

(4) $$\bar{S}\left(X,Y\right)Z=\frac{1}{2}\left\{\bar{R}\left(X,Y\right)Z+{\bar{R}}^{*}\left(X,Y\right)Z\right\},$$

for any $X,Y,Z,W\in \Gamma (T\bar{M})$.

Set a tensor field $\bar{K}\in \Gamma \left(T{\bar{M}}^{\left(1,2\right)}\right)$ by:

(5) $${\bar{K}}_{X}Y=\bar{K}\left(X,Y\right)={\bar{\nabla }}_{X}Y-{\bar{\nabla }}_{X}^{0}Y.$$

Furthermore, we have:

$$\bar{K}\left(X,Y\right)={\bar{\nabla }}_{X}^{0}Y-{\bar{\nabla }}_{X}^{*}Y=\frac{1}{2}\left({\bar{\nabla }}_{X}Y-{\bar{\nabla }}_{X}^{*}Y\right).$$

Then $\bar{K}$ has the properties:

$$\bar{K}\left(X,Y\right)=\bar{K}\left(Y,X\right),$$

$$g\left(\bar{K}\left(X,Y\right),Z\right)=g\left(Y,\bar{K}\left(X,Z\right)\right).$$

Next, we consider $\left(\bar{M},g,\phi ,\xi \right)$ a (2n+1)-dimensional Kenmotsu manifold defined as an almost contact metric manifold $\bar{M}$ which satisfies for any $X,Y\in \Gamma (T\bar{M})$ the relations:

$$\left({\bar{\nabla }}_{X}^{0}\phi \right)\left(Y\right)=g\left(\phi X,Y\right)\xi -\bar{\eta }\left(Y\right)\phi X,$$

$${\bar{\nabla }}_{X}^{0}\xi =X-\bar{\eta }\left(X\right)\xi ,$$

where $\phi \in \Gamma (T{\bar{M}}^{\left(1,1\right)})$, $\xi \in \Gamma (T\bar{M})$, $\bar{\eta }$ is a 1-form on $\bar{M}$ with $\bar{\eta }\left(X\right)=g\left(X,\xi \right)$.

A Kenmotsu manifold $\bar{M}$ with a statistical structure $\left(\bar{\nabla },g\right)$ is called a Kenmotsu statistical manifold [25] if the following formula holds for any $X,Y\in \Gamma (T\bar{M})$:

$$\bar{K}\left(X,\phi Y\right)=-\phi \bar{K}\left(X,Y\right),$$

where $\bar{K}$ is the tensor field defined in (5),

A Kenmotsu statistical manifold ($\bar{M},\bar{\nabla },g,\phi ,\xi$) is said to be of constant ϕ-sectional curvature *c* if and only if [25]:

(6) $$\begin{array}{ccc} \bar{S}\left(X,Y\right)Z & = & \frac{c-3}{4}\left\{g\left(Y,Z\right)X-g\left(X,Z\right)Y\right)\}\\ & + & \frac{c+1}{4}\{g\left(\phi Y,Z\right)\phi X-g\left(\phi X,Z\right)\phi Y-2g\left(\phi X,Y\right)\phi Z\\ & - & g(Y,\xi )g(Z,\xi )X+g(X,\xi )g(Z,\xi )Y\\ & + & g(Y,\xi )g(Z,X)\xi -g(X,\xi )g(Z,Y)\xi \}, \end{array}$$

for any $X,Y,Z\in \Gamma (T\bar{M})$.

On the other hand, assume that $\tilde{\nabla }$ is a linear connection on $\bar{M}$. Then $\tilde{\nabla }$ is called a *semi-symmetric connection* if the torsion tensor $\tilde{T}$ of $\tilde{\nabla }$ defined by

$$\tilde{T}\left(X,Y\right)={\tilde{\nabla }}_{X}Y-{\tilde{\nabla }}_{Y}X-\left[X,Y\right]$$

satisfies for any $X,Y\in \Gamma (T\bar{M})$ the relation:

(7) $$\tilde{T}\left(X,Y\right)=\eta \left(Y\right)X-\eta \left(X\right)Y,$$

where η is a 1-form. Moreover, the connection $\tilde{\nabla }$ is called a *semi-symmetric metric connection* on $\bar{M}$ if we have $\tilde{\nabla }g=0$ (see [27]).

Next, we will denote by γ the (1,2)-tensor field defined by

$$\gamma \left(X,Y\right)=\left({\bar{\nabla }}_{X}^{0}\eta \right)Y-\left(\nabla_{X}^{0}\eta \right)Y.$$

Let $\left(\bar{M},g,\bar{\nabla }\right)$ be a statistical manifold endowed with a semi-symmetric metric connection $\tilde{\nabla }$. Then $\tilde{\nabla }$ satisfies for any $X,Y\in \Gamma (T\bar{M})$ [39]:

(8) $${\tilde{\nabla }}_{X}Y={\bar{\nabla }}_{X}Y+\eta \left(Y\right)X-g\left(X,Y\right)U-{\bar{K}}_{X}Y,$$

(9) $${\tilde{\nabla }}_{X}Y={\bar{\nabla }}_{X}^{*}Y+\eta \left(Y\right)X-g\left(X,Y\right)U-{\bar{K}}_{X}Y,$$

where *U* is a vector field such that g(U,X)=η(X), $\bar{K}$ is the difference tensor field defined in (5).

Let *M* be an (m+1)-dimensional submanifold of a statistical manifold $\bar{M}$ endowed with a semi-symmetric metric connection $\tilde{\nabla }$. Denote $\nabla^{\prime }$ the induced connection and $h^{\prime }$ the second fundamental form on *M* with respect to $\tilde{\nabla }$. Then the Gauss formula with respect to $\tilde{\nabla }$ is:

(10) $${\tilde{\nabla }}_{X}Y=\nabla_{X}^{\prime }Y+h^{\prime }\left(X,Y\right).$$

In addition, the Gauss equation with respect to $\tilde{\nabla }$ is [39]:

(11) $$\begin{array}{c} g\left(\tilde{R}\left(X,Y\right)Z,W\right)=g\left(R^{\prime }\left(X,Y\right)Z,W\right)+g\left(h^{\prime }\left(X,Z\right),h^{\prime }\left(Y,W\right)\right)\\ -g(h^{\prime }\left(Y,Z\right),h^{\prime }\left(X,W\right)), \end{array}$$

where $\tilde{R}$ and $R^{\prime }$ are the curvature tensor fields associated with the connections $\tilde{\nabla }$ and $\nabla^{\prime }$, respectively.

We notice that $h^{\prime }$ coincides with the second fundamental form of the Levi-Civita connection (see, e.g., [39]). Thus, $h^{\prime }$ becomes:

(12) $$h^{\prime }\left(X,Y\right)=\frac{1}{2}\left(h\left(X,Y\right)+h^{*}\left(X,Y\right)\right).$$

According to Kazan et al. [38], the relations between the curvature tensor $\tilde{R}$ of $\tilde{\nabla }$ and the curvature tensors $\bar{R}$ and ${\bar{R}}^{*}$ of the connections $\bar{\nabla }$ and ${\bar{\nabla }}^{*}$ are as follows:

(13) $$\begin{array}{c} \tilde{R}\left(X,Y\right)Z=\bar{R}\left(X,Y\right)Z+\left\{\eta \left(X\right)U-\eta \left(U\right)X-{\bar{\nabla }}_{X}U+\bar{K}\left(X,U\right)\right\}g\left(Y,Z\right)\\ +\{\eta \left(Y\right)U-\eta \left(U\right)Y-{\bar{\nabla }}_{Y}U+\bar{K}\left(Y,U\right)\}g\left(X,Z\right)\\ -g\left(\eta \left(X\right)U-{\bar{\nabla }}_{X}U+\bar{K}\left(X,U\right),Z\right)Y+g\left(\eta \left(Y\right)U-{\bar{\nabla }}_{Y}U+\bar{K}\left(Y,U\right),Z\right)X-\\ \left({\bar{\nabla }}_{X}\bar{K}\right)\left(Y,Z\right)+\left({\bar{\nabla }}_{Y}\bar{K}\right)\left(X,Z\right)+\bar{K}\left(X,\bar{K}\left(Y,Z\right)\right)-\bar{K}\left(Y,\bar{K}\left(X,Z\right)\right), \end{array}$$

and

(14) $$\begin{array}{c} \tilde{R}\left(X,Y\right)Z={\bar{R}}^{*}\left(X,Y\right)Z+\left\{\eta \left(X\right)U-\eta \left(U\right)X-{\bar{\nabla }}_{X}^{*}U-\bar{K}\left(X,U\right)\right\}g\left(Y,Z\right)\\ -\{\eta \left(Y\right)U-\eta \left(U\right)Y-{\bar{\nabla }}_{Y}^{*}U-\bar{K}\left(Y,U\right)\}g\left(X,Z\right)\\ -g\left(\eta \left(X\right)U-{\bar{\nabla }}_{X}^{*}U-\bar{K}\left(X,U\right),Z\right)Y+g\left(\eta \left(Y\right)U-{\bar{\nabla }}_{Y}^{*}U-\bar{K}\left(Y,U\right),Z\right)X+\\ \left({\bar{\nabla }}_{X}^{*}\bar{K}\right)\left(Y,Z\right)-\left({\bar{\nabla }}_{Y}\bar{K}\right)\left(X,Z\right)+\bar{K}\left(X,\bar{K}\left(Y,Z\right)\right)-\bar{K}\left(Y,\bar{K}\left(X,Z\right)\right), \end{array}$$

for any $X,Y,Z\in \Gamma (T\bar{M})$.

On the other hand, since the induced connection $\nabla^{\prime }$ of the semi-symmetric metric connection $\tilde{\nabla }$ is also semi-symmetric metric connection [39], then the Gauss formula (10) becomes:

(15) $$\begin{array}{c} g\left(\tilde{R}\left(X,Y\right)Z,W\right)=g\left(R\left(X,Y\right)Z,W\right)\;\\ +\left\{\eta \left(X\right)\eta \left(W\right)-\eta \left(U\right)g\left(X,W\right)-g\left(\nabla_{X}U,W\right)+g\left(K_{X}U,W\right)\right\}g\left(Y,Z\right)\\ +\left\{\eta \left(Y\right)\eta \left(W\right)-\eta \left(U\right)g\left(Y,W\right)-g\left(\nabla_{Y}U,W\right)+g\left(K_{Y}U,W\right)\right\}g\left(X,Z\right)\\ -g\left(\eta \left(X\right)U-\nabla_{X}U+K\left(X,U\right),Z\right)g\left(Y,W\right)+g\left(\eta \left(Y\right)U-\nabla_{Y}U+K\left(Y,U\right),Z\right)g\left(X,W\right)\\ -g\left(\left(\nabla_{X}K\right)\left(Y,Z\right),W\right)+g\left(\left(\nabla_{Y}K\right)\left(X,Z\right),W\right)+g\left(K_{X}K\left(Y,Z\right),W\right)-g\left(K_{Y}K\left(X,Z\right),W\right)\\ -\frac{1}{4}g\left(h\left(X,W\right)+h^{*}\left(X,W\right),h\left(Y,Z\right)+h^{*}\left(Y,Z\right)\right)\\ +\frac{1}{4}g\left(h\left(X,Z\right)+h^{*}\left(X,Z\right),h\left(Y,W\right)+h^{*}\left(Y,W\right)\right), \end{array}$$

where $K_{X}Y=\frac{1}{2}\left(\nabla -\nabla^{*}\right)$ and *R* is the curvature tensor of the induced statistical connection ∇ on the submanifold *M*.

Similarly, we can obtain the Gauss formula involving the curvature tensor $R^{*}$ of the induced statistical connection $\nabla^{*}$ on *M* as follows:

(16) $$\begin{array}{c} g\left(\tilde{R}\left(X,Y\right)Z,W\right)=g\left(R^{*}\left(X,Y\right)Z,W\right)\;\\ +\left\{\eta \left(X\right)\eta \left(W\right)-\eta \left(U\right)g\left(X,W\right)-g\left(\nabla_{X}^{*}U,W\right)+g\left(K_{X}U,W\right)\right\}g\left(Y,Z\right)\\ +\left\{\eta \left(Y\right)\eta \left(W\right)-\eta \left(U\right)g\left(Y,W\right)-g\left(\nabla_{Y}^{*}U,W\right)+g\left(K_{Y}U,W\right)\right\}g\left(X,Z\right)\\ -g\left(\eta \left(X\right)U-\nabla_{X}^{*}U+K\left(X,U\right),Z\right)g\left(Y,W\right)+g\left(\eta \left(Y\right)U-\nabla_{Y}^{*}U+K\left(Y,U\right),Z\right)g\left(X,W\right)\\ -g\left(\left(\nabla_{X}^{*}K\right)\left(Y,Z\right),W\right)+g\left(\left(\nabla_{Y}^{*}K\right)\left(X,Z\right),W\right)+g\left(K_{X}K\left(Y,Z\right),W\right)-g\left(K_{Y}K\left(X,Z\right),W\right)\\ -\frac{1}{4}g\left(h\left(X,W\right)+h^{*}\left(X,W\right),h\left(Y,Z\right)+h^{*}\left(Y,Z\right)\right)\\ +\frac{1}{4}g\left(h\left(X,Z\right)+h^{*}\left(X,Z\right),h\left(Y,W\right)+h^{*}\left(Y,W\right)\right). \end{array}$$

If x∈M and $\pi \subset T_{x}M$ is a non-degenerate 2-plane, then the *sectional curvature* σ is defined as [40]:

(17) $$\begin{array}{c} \sigma \left(\pi \right)=\sigma \left(X\wedge Y\right)=\frac{g(S(X,Y)Y,X)}{g\left(X,X\right)g\left(Y,Y\right)-g^{2}\left(X,Y\right)}, \end{array}$$

where {X,Y} is a basis of π.

The *scalar curvature* τ of (M,∇,g) at a point x∈M is defined by:

(18) $$\begin{array}{c} \tau \left(x\right)=\sum_{1\leq i<j\leq m+1}\sigma \left(e_{i}\wedge e_{j}\right)=\sum_{1\leq i<j\leq m+1}g\left(S\left(e_{i},e_{j}\right)e_{j},e_{i}\right), \end{array}$$

where $\left\{e_{1},...,e_{m+1}\right\}$ is an orthonormal basis at *x*. On the other hand, the normalized scalar curvature ρ of (M,∇,g) at a point x∈M is given by

(19) $$\rho \left(x\right)=\frac{2\tau (x)}{m(m+1)}.$$

The *mean curvature* vector fields of *M* are defined by, respectively:

$$H=\frac{1}{m+1}\sum_{i=1}^{m+1}h\left(e_{i},e_{i}\right),\;\;H^{*}=\frac{1}{m+1}\sum_{i=1}^{m+1}h^{*}\left(e_{i},e_{i}\right).$$

It follows that we have $2h^{0}=h+h^{*}$ and $2H^{0}=H+H^{*}$, where $h^{0}$ and $H^{0}$ are the second fundamental form and the mean curvature field of *M*, respectively, with respect to the Levi–Civita connection $\nabla^{0}$ on *M*.

Then, the *squared mean curvatures* of the submanifold *M* in $\bar{M}$ are given by:

$${∥H∥}^{2}=\frac{1}{{\left(m+1\right)}^{2}}\sum_{\alpha =m+2}^{2n+1}{\left(\sum_{i=1}^{m+1}h_{ii}^{\alpha }\right)}^{2},\;\;{∥H^{*}∥}^{2}=\frac{1}{{\left(m+1\right)}^{2}}\sum_{\alpha =m+2}^{2n+1}{\left(\sum_{i=1}^{m+1}h_{ii}^{*\alpha }\right)}^{2},$$

where $h_{ij}^{\alpha }=g\left(h\left(e_{i},e_{j}\right),e_{\alpha }\right)$ and $h_{ij}^{*\alpha }=g\left(h^{*}\left(e_{i},e_{j}\right),e_{\alpha }\right)$, for i,j∈{1,...,m+1}, α∈{m+2,...,2n+1}.

The *Casorati curvatures* of the submanifold *M* in $\bar{M}$ are defined by the squared norms of *h* and $h^{*}$ over the dimension (m+1), denoted by C and $C^{*}$, respectively, as follows:

$$C=\frac{1}{m+1}{∥h∥}^{2}=\frac{1}{m+1}\sum_{\alpha =m+2}^{2n+1}\sum_{i,j=1}^{m+1}{\left(h_{ij}^{\alpha }\right)}^{2},$$

$$C^{*}=\frac{1}{m+1}{∥h^{*}∥}^{2}=\frac{1}{m+1}\sum_{\alpha =m+2}^{2n+1}\sum_{i,j=1}^{m+1}{\left(h_{ij}^{*\alpha }\right)}^{2},$$

where $h_{ij}^{\alpha }$ and $h_{ij}^{*\alpha }$ are defined above.

Let *L* be an *s*-dimensional subspace of $T_{x}M$, s≥2 and let $\left\{e_{1},\ldots ,e_{s}\right\}$ be an orthonormal basis of *L*. Then the Casorati curvatures C(L) and $C^{*}\left(L\right)$ of *L* are given by:

$$C\left(L\right)=\frac{1}{s}\sum_{\alpha =m+2}^{2n+1}\sum_{i,j=1}^{s}{\left(h_{ij}^{\alpha }\right)}^{2},\;\;C^{*}\left(L\right)=\frac{1}{s}\sum_{\alpha =m+2}^{2n+1}\sum_{i,j=1}^{s}{\left(h_{ij}^{*\alpha }\right)}^{2}.$$

The *normalized δ-Casorati curvatures* $\delta_{C}\left(m\right)$ and ${\hat{\delta }}_{C}\left(m\right)$ of the submanifold *M* are given by:

$$\delta_{C}{\left(m\right)|}_{x}=\frac{1}{2}C{|}_{x}+\frac{m+2}{2(m+1)}\inf\left\{C\left(L\right)|L\;a\;\mathrm{hyperplane}\;\mathrm{of}\;T_{x}M\right\}$$

and

$${\hat{\delta }}_{C}{\left(m\right)|}_{x}=2C{|}_{x}-\frac{2m+1}{2(m+1)}\sup\left\{C\left(L\right)|L\;a\;\mathrm{hyperplane}\;\mathrm{of}\;T_{x}M\right\}.$$

Furthermore, the dual normalized $\delta^{*}$-Casorati curvatures $\delta_{C}^{*}\left(m\right)$ and ${\hat{\delta }}_{C}^{*}\left(m\right)$ of the submanifold *M* in $\bar{M}$ are defined as follows:

$$\delta_{C}^{*}{\left(m\right)|}_{x}=\frac{1}{2}C^{*}{|}_{x}+\frac{m+2}{2(m+1)}\inf\left\{C^{*}\left(L\right)|L\;a\;\mathrm{hyperplane}\;\mathrm{of}\;T_{x}M\right\}$$

and

$${\hat{\delta }}_{C}^{*}{\left(m\right)|}_{x}=2C^{*}{|}_{x}-\frac{2m+1}{2(m+1)}\sup\left\{C^{*}\left(L\right)|L\;a\;\mathrm{hyperplane}\;\mathrm{of}\;T_{x}M\right\}.$$

The *generalized normalized δ-Casorati curvatures* $\delta_{C}\left(r;m\right)$ and ${\hat{\delta }}_{C}\left(r;m\right)$ of *M* in $\bar{M}$ are defined in [13] by:

$$\delta_{C}{\left(r;m\right)|}_{x}=r\;C{|}_{x}+a\left(r\right)\;\inf\left\{C\left(L\right)|L\;a\;\mathrm{hyperplane}\;\mathrm{of}\;T_{x}M\right\},$$

if 0<r<m(m+1), and

$${\hat{\delta }}_{C}{\left(r;m\right)|}_{x}=r\;C{|}_{x}+a\left(r\right)\;\sup\left\{C\left(L\right)|L\;a\;\mathrm{hyperplane}\;\mathrm{of}\;T_{x}M\right\},$$

if r>m(m+1), where a(r) is set as

$$\begin{array}{c} a\left(r\right)=\frac{m\left(r+m+1\right)\left(m^{2}+m-r\right)}{(m+1)r}, \end{array}$$

for any positive real number *r*, different from m(m+1).

Moreover, the dual generalized normalized $\delta^{*}$-Casorati curvatures $\delta_{C}^{*}\left(r;m\right)$ and ${\hat{\delta^{*}}}_{C}\left(r;m\right)$ of the submanifold *M* in $\bar{M}$ are given by:

$$\delta_{C}^{*}{\left(r;m\right)|}_{x}=r\;C^{*}{|}_{x}+a\left(r\right)\;\inf\left\{C^{*}\left(L\right)|L\;a\;\mathrm{hyperplane}\;\mathrm{of}\;T_{x}M\right\},$$

if 0<r<m(m+1), and

$${\hat{\delta }}_{C}^{*}{\left(r;m\right)|}_{x}=r\;C^{*}{|}_{x}+a\left(r\right)\;\sup\left\{C^{*}\left(L\right)|L\;a\;\mathrm{hyperplane}\;\mathrm{of}\;T_{x}M\right\},$$

if r>m(m+1), where a(r) is expressed above.

Next, we consider the following constrained extremum problem

(20) $$\min_{x\in M}f\left(x\right),$$

where *M* is a submanifold of a Riemannian manifold $\left(\bar{M},g\right)$, and $f:\bar{M}\to R$ is a function of differentiability class $C^{2}$. In this setting, we recall the following result which we will use later.

**Theorem 1**  ([42])**.** *If the Riemannian submanifold M is complete and connected, $\left(gradf\right)\left(x_{0}\right)\in T_{x_{0}}^{⊥}M$ for a point $x_{0}\in M$, and the bilinear form $V:T_{x_{0}}M\times T_{x_{0}}M\to R$ defined by:*

(21) $$V\left(X,Y\right)=\mathrm{Hess}\left(f\right)\left(X,Y\right)+g(\hat{h}\left(X,Y\right),\mathrm{grad}f),$$

*is positive definite in $x_{0}$, then $x_{0}$ is the optimal solution of the problem *(20)*, where $\hat{h}$ is the second fundamental form of M.*

**Remark** **1.** *If the bilinear form V defined by *(21)* is positive semi-definite on the submanifold M, then the critical points of f|M are global optimal solutions of the problem *(20)*. For more details see *([43], *Remark 3.2).*

## 3. Main Inequalities

**Theorem** **2.** *Let ($\bar{M},\bar{\nabla },g,\phi ,\xi$) be a (2n+1)-dimensional Kenmotsu statistical manifold of constant ϕ-sectional curvature c, endowed with a semi-symmetric metric connection $\tilde{\nabla }$. Suppose M is an (m+1)-dimensional statistical submanifold of ($\bar{M},\bar{\nabla },g,\phi ,\xi$) such that ξ is a tangent vector field on M. Then the generalized normalized δ-Casorati curvatures fulfill the following inequalities:*

*(i)*  (22) $$\begin{array}{c} \delta_{C}^{0}\left(r;m\right)\geq 2\tau -\frac{c-3}{4}m\left(m+1\right)-\frac{3(c+1)}{4}{∥P∥}^{2}+\frac{1}{2}m\left(c+1\right)+2m\;trace\left(\gamma \right) \end{array}$$
  for any r∈R with 0<r<m(m+1), where $\delta_{C}^{0}\left(r,m\right)$ is defined by $2\delta_{C}^{0}\left(r,m\right)=\delta_{C}\left(r;m\right)+\delta_{C}^{*}\left(r;m\right)$, and
*(ii)*  (23) $$\begin{array}{c} {\hat{\delta }}_{C}^{0}\left(r;m\right)\geq 2\tau -\frac{c-3}{4}m\left(m+1\right)-\frac{3(c+1)}{4}{∥P∥}^{2}+\frac{1}{2}m\left(c+1\right)+2m\;trace\left(\gamma \right) \end{array}$$
  for any r∈R with r>m(m+1), where ${\hat{\delta }}_{C}^{0}\left(r,m\right)$ is defined by $2{\hat{\delta }}_{C}^{0}\left(r,m\right)={\hat{\delta }}_{C}\left(r;m\right)+{\hat{\delta }}_{C}^{*}\left(r;m\right).$

*Furthermore, the case of equality of any of the inequalities *(22) *and* (23) *holds at all points p∈M if and only if:*

$$\begin{array}{c} h=-h^{*}. \end{array}$$

**Proof.**  From Equations (13) and (14), we obtain:

(24) $$\begin{array}{c} \tilde{R}\left(X,Y\right)Z=\bar{S}\left(X,Y\right),Z+\left\{\eta \left(X\right)U-\eta \left(U\right)X-{\bar{\nabla }}_{X}^{0}U\right\}g\left(Y,Z\right)\\ -\{\eta \left(Y\right)U-\eta \left(U\right)Y-{\bar{\nabla }}_{Y}^{0}U\}g\left(X,Z\right)\\ -g\left(\eta \left(X\right)U-{\bar{\nabla }}_{X}^{0}U,Z\right)Y+g\left(\eta \left(Y\right)U-{\bar{\nabla }}_{Y}^{0}U,Z\right)X\\ -\frac{1}{2}\left[\left({\bar{\nabla }}_{X}\bar{K}\right)\left(Y,Z\right)-\left({\bar{\nabla }}_{X}^{*}\bar{K}\right)\left(Y,Z\right)\right]+\frac{1}{2}\left[\left({\bar{\nabla }}_{Y}\bar{K}\right)\left(X,Z\right)-\left({\bar{\nabla }}_{Y}^{*}\bar{K}\right)\left(X,Z\right)\right]\\ +\bar{K}\left(X,\bar{K}\left(Y,Z\right)\right)-\bar{K}\left(Y,\bar{K}\left(X,Z\right)\right). \end{array}$$

Moreover, using the definition (5), the formula (24) becomes:

(25) $$\begin{array}{c} \tilde{R}\left(X,Y\right)Z=\bar{S}\left(X,Y\right),Z+\left\{\eta \left(X\right)U-\eta \left(U\right)X-{\bar{\nabla }}_{X}^{0}U\right\}g\left(Y,Z\right)\\ -\{\eta \left(Y\right)U-\eta \left(U\right)Y-{\bar{\nabla }}_{Y}^{0}U\}g\left(X,Z\right)\\ -g\left(\eta \left(X\right)U-{\bar{\nabla }}_{X}^{0}U,Z\right)Y+g\left(\eta \left(Y\right)U-{\bar{\nabla }}_{Y}^{0}U,Z\right)X. \end{array}$$

Next, the relation (25) implies:

(26) $$\begin{array}{c} g\left(\tilde{R}\left(X,Y\right)Z,W\right)=g\left(\bar{S}\left(X,Y\right)Z,W\right)-\lambda \left(Y,Z\right)g\left(X,W\right)\\ +\lambda (X,Z)g(Y,W)-\lambda (X,W)g(Y,Z)+\lambda (Y,W)g(X,Z), \end{array}$$

for any $X,Y,Z,W\in \Gamma (T\bar{M})$, where λ is expressed by:

$$\lambda \left(X,Y\right)=\left({\bar{\nabla }}_{X}^{0}\eta \right)Y-\eta \left(X\right)\eta \left(Y\right)+\frac{1}{2}\eta \left(U\right)g\left(X,Y\right).$$

On the other hand, from the formula (6), we obtain:

(27) $$\begin{array}{ccc} g(\bar{S}\left(X,Y\right)Z,W) & = & \frac{c-3}{4}\left\{g\left(Y,Z\right)g\left(X,W\right)-g\left(X,Z\right)g\left(Y,W\right)\right\}\\ & + & \frac{c+1}{4}\{g\left(\phi Y,Z\right)g\left(\phi X,W\right)-g\left(\phi X,Z\right)g\left(\phi Y,W\right)\\ & - & 2g(\phi X,Y)g(\phi Z,W)-g(Y,\xi )g(Z,\xi )g(X,W)+g(X,\xi )g(Z,\xi )g(Y,W)\\ & + & g(Y,\xi )g(Z,X)g(\xi ,W)-g(X,\xi )g(Z,Y)g(\xi ,W)\}, \end{array}$$

for any $X,Y,Z,W\in \Gamma (T\bar{M})$.

For x∈M, let $\left\{e_{1},...,e_{m+1}=\xi \right\}$ and $\left\{e_{m+2},...,e_{2n+1}\right\}$ be orthonormal bases of $T_{x}M$ and $T_{x}^{⊥}M$, respectively. Suppose $X=W=e_{i}$ and $Y=Z=e_{j}$ (i≠j, with i,j∈{1,...,m+1}) in the relations (26) and (27), then we obtain:

(28) $$\begin{array}{c} g\left(\tilde{R}\left(e_{i},e_{j}\right)e_{j},e_{i}\right)=\frac{c-3}{4}+\frac{c+1}{4}\left\{3g^{2}\left(\phi e_{i},e_{j}\right)-g^{2}\left(e_{j},\xi \right)-g^{2}\left(e_{i},\xi \right)\right\}\\ -\lambda \left(e_{i},e_{i}\right)-\lambda \left(e_{j},e_{j}\right). \end{array}$$

On the other hand, from the Gauss formulas (15) and (16) we obtain:

(29) $$\begin{array}{c} g\left(\tilde{R}\left(X,Y\right)Z,W\right)=g\left(S\left(X,Y\right)Z,W\right)-\mu \left(Y,Z\right)g\left(X,W\right)\\ +\mu (X,Z)g(Y,W)-\mu (X,W)g(Y,Z)+\mu (Y,W)g(X,Z)\\ -\frac{1}{4}g\left(h\left(X,W\right)+h^{*}\left(X,W\right),h\left(Y,Z\right)+h^{*}\left(Y,Z\right)\right)\\ +\frac{1}{4}g\left(h\left(X,Z\right)+h^{*}\left(X,Z\right),h\left(Y,W\right)+h^{*}\left(Y,W\right)\right), \end{array}$$

for any X,Y,Z,W∈Γ(TM), where μ has the following expression:

$$\mu \left(X,Y\right)=\left(\nabla_{X}^{0}\eta \right)Y-\eta \left(X\right)\eta \left(Y\right)+\frac{1}{2}\eta \left(U\right)g\left(X,Y\right),$$

with $\nabla^{0}=\frac{\nabla +\nabla^{*}}{2}$. Now, we can easily see that we have

$$\lambda \left(X,Y\right)-\mu \left(X,Y\right)=\left({\bar{\nabla }}_{X}^{0}\eta \right)Y-\left(\nabla_{X}^{0}\eta \right)Y=\gamma \left(X,Y\right),$$

for any X,Y∈Γ(TM).

For $X=W=e_{i}$ and $Y=Z=e_{j}$, from (29) we have:

(30) $$\begin{array}{c} g\left(\tilde{R}\left(e_{i},e_{j}\right)e_{j},e_{i}\right)=g(S\left(\left(e_{i},e_{j}\right)e_{j},e_{i}\right)-\mu \left(e_{i},e_{i}\right)-\mu \left(e_{j},e_{j}\right)\\ -\frac{1}{4}g(h\left(e_{i},e_{i}\right)+h^{*}\left(e_{i},e_{i}\right),h\left(e_{j},e_{j}\right)+h^{*}\left(e_{j},e_{j}\right)\\ +\frac{1}{4}g\left(h\left(e_{i},e_{j}\right)+h^{*}\left(e_{i},e_{j}\right),h\left(e_{j},e_{i}\right)+h^{*}\left(e_{j},e_{i}\right)\right). \end{array}$$

Next, from (28) and (30) it follows that:

(31) $$\begin{array}{c} \frac{c-3}{4}+\frac{c+1}{4}\left\{3g^{2}\left(\phi e_{i},e_{j}\right)-g^{2}\left(e_{i},\xi \right)-g^{2}\left(e_{j},\xi \right)\right\}\\ -\lambda \left(e_{i},e_{i}\right)-\lambda \left(e_{j},e_{j}\right)+\mu \left(e_{i},e_{i}\right)+\mu \left(e_{j},e_{j}\right)=\\ g\left(S\left(\left(e_{i},e_{j}\right)e_{j},e_{i}\right)\right)-\frac{1}{4}g\left(h\left(e_{i},e_{i}\right)+h^{*}\left(e_{i},e_{i}\right),h\left(e_{j},e_{j}\right)+h^{*}\left(e_{j},e_{j}\right)\right)\\ +\frac{1}{4}g\left(h\left(e_{i},e_{j}\right)+h^{*}\left(e_{i},e_{j}\right),h\left(e_{j},e_{i}\right)+h^{*}\left(e_{j},e_{i}\right)\right). \end{array}$$

We remind that any vector field X∈Γ(TM) admits a unique decomposition into its tangent and normal components PX and PY, respectively, as follows:

ϕX=PX+FX.

Next, by summation over 1≤i,j≤m+1, Equation (31) becomes:

(32) $$\begin{array}{c} \frac{c-3}{4}m\left(m+1\right)+\frac{c+1}{4}{(3∥P∥}^{2}-2m)-2m\;trace\left(\gamma \right)=\\ 2\tau +\frac{1}{4}\left(m+1\right)C^{0}-{\left(m+1\right)}^{2}{∥H^{0}∥}^{2}, \end{array}$$

where ${∥P∥}^{2}$ is the squared norm of *P* expressed by

$${∥P∥}^{2}=\sum_{1\leq i,j\leq m+1}g^{2}\left(Pe_{i},e_{j}\right).$$

Let P be a quadratic polynomial in the components of the second fundamental form given by:

(33) $$\begin{array}{c} P=rC^{0}+a\left(r\right)C^{0}\left(L\right)+\frac{c-3}{4}m\left(m-1\right)+\\ \frac{3(c+1)}{4}{∥P∥}^{2}-\frac{1}{2}m\left(c+1\right)-2m\;trace\left(\gamma \right)-2\tau . \end{array}$$

We will prove that P≥0.

Consider, without loss of generality, that *L* is spanned by $e_{1},e_{2},...,e_{m}$. Then, the expression of P in (33) becomes:

$$\begin{array}{c} P=\left(r+m+1\right)C^{0}+a\left(r\right)C^{0}\left(L\right)-{\left(m+1\right)}^{2}{∥H^{0}∥}^{2}. \end{array}$$

Moreover, the above relation implies:

(34) $$\begin{array}{c} P=\sum_{\alpha =m+2}^{2n+1}\left[\frac{m+r+1}{m+1}\sum_{i,j=1}^{m+1}{\left(h_{ij}^{0\alpha }\right)}^{2}+\frac{a(r)}{m}\sum_{i,j=1}^{m}{\left(h_{ij}^{0\alpha }\right)}^{2}-{\left(\sum_{i=1}^{m+1}h_{ii}^{0\alpha }\right)}^{2}\right]. \end{array}$$

Furthermore, P given by (34) can be written as:

$$\begin{array}{ccc} P & = & \sum_{\alpha =m+2}^{2n+1}\{\left[\frac{2(m+r+1)}{m+1}+\frac{2a(r)}{m}\right]\sum_{1\leq i<j\leq m}{\left(h_{ij}^{0\alpha }\right)}^{2}+\frac{2(m+r+1)}{m+1}\sum_{i=1}^{m}{\left(h_{i\;m+1}^{0\alpha }\right)}^{2}\\ & & +\left(\frac{m+r+1}{m+1}+\frac{a(r)}{m}-1\right)\sum_{i=1}^{m}{\left(h_{ii}^{0\alpha }\right)}^{2}-2\sum_{1\leq i<j\leq m+1}h_{ii}^{0\alpha }h_{jj}^{0\alpha }+\frac{r}{m+1}{\left(h_{m+1\;m+1}^{0\alpha }\right)}^{2}\}. \end{array}$$

The latter equation implies:

$$\begin{array}{ccc} P & \geq & \sum_{\alpha =m+2}^{2n+1}\left[\frac{rm+a(r)(m+1)}{m(m+1)}\sum_{i=1}^{m}{\left(h_{ii}^{0\alpha }\right)}^{2}+\frac{r}{m+1}{\left(h_{m+1\;m+1}^{0\alpha }\right)}^{2}-2\sum_{1\leq i<j\leq m+1}h_{ii}^{0\alpha }h_{jj}^{0\alpha }\right]. \end{array}$$

Now, suppose that $f_{\alpha }$ is a quadratic form expressed by $f_{\alpha }:R^{m+1}\to R$, for α∈{m+2,...,2n+1}:

$$\begin{array}{ccc} f_{\alpha }\left(h_{11}^{0\alpha },h_{22}^{0\alpha },...,h_{m+1\;m+1}^{0\alpha }\right) & = & \frac{mr+(m+1)a(r)}{m(m+1)}\sum_{i=1}^{m}{\left(h_{ii}^{0\alpha }\right)}^{2}\\ & & +\frac{r}{m+1}{\left(h_{m+1\;m+1}^{0\alpha }\right)}^{2}-2\sum_{1\leq i<j\leq m+1}h_{ii}^{0\alpha }h_{jj}^{0\alpha }. \end{array}$$

Our aim is to investigate the constrained extremum problem

$$\min f_{\alpha }$$

under the constraint

(35) $$Q:h_{11}^{0\alpha }+h_{22}^{0\alpha }+...+h_{m+1\;m+1}^{0\alpha }=k^{\alpha },$$

where $k^{\alpha }$ is a real constant. In this respect, we establish the following first order partial derivatives system:

$$\left\{\begin{array}{c} \frac{\partial f_{\alpha }}{\partial h_{ii}^{0\alpha }}=\frac{2[mr+(m+1)(a(r)+m)]}{m(m+1)}h_{ii}^{0\alpha }-2\sum_{k=1}^{m+1}h_{kk}^{0\alpha }=0\\ \frac{\partial f_{\alpha }}{\partial h_{m+1\;m+1}^{0\alpha }}=\frac{2r}{m+1}h_{m+1\;m+1}^{0\alpha }-2\sum_{k=1}^{m}h_{kk}^{0\alpha }=0, \end{array}\right.$$

for all i∈{1,...,m}, α∈{m+2,...,2n+1}.

By using the constraint *Q* defined by (35), the above system provides the critical point:

$$h_{ii}^{0\alpha }=\frac{m\left(m+1\right)k^{\alpha }}{\left(m+1)a(r)+mr+m(m+1\right)},$$

$$h_{m+1\;m+1}^{0\alpha }=\frac{\left(m+1\right)k^{\alpha }}{m+r+1},$$

for all i∈{1,...,m}, α∈{m+2,...,2n+1}.

For x∈ Q, we define the 2-form $V:T_{x}Q\times T_{x}Q\to R$ by:

$$V\left(X,Y\right)=\mathrm{Hess}\left(f_{\alpha }\right)\left(X,Y\right)+\langle \hat{h}\left(X,Y\right),\left(\mathrm{grad}f_{\alpha }\right)\left(x\right)\rangle ,$$

where $\hat{h}$ denotes the second fundamental form of *Q* in $R^{m+1}$ and 〈·,·〉 stands for the standard inner product on $R^{m+1}$.

We achieve also the Hessian matrix of $f_{\alpha }$ with the expression:

$$\mathrm{Hess}\left(f_{\alpha }\right)=\left(\begin{array}{ccccc} \beta & -2 & \cdots & -2 & -2\\ -2 & \beta & \cdots & -2 & -2\\ \vdots & \vdots & \ddots & \vdots & \vdots \\ -2 & -2 & \cdots & \beta & -2\\ -2 & -2 & \cdots & -2 & \frac{2r}{m+1} \end{array}\right),$$

where β is a real constant set as $\beta =\frac{2[mr+(m+1)a(r)]}{m(m+1)}$.

Assume that $X=(X_{1},...,X_{m+1})$ is a tangent vector field to the hyperplane *Q* at *x* such that $\sum_{i=1}^{m+1}X_{i}=0$. Then we have:

(36) $$V\left(X,X\right)=\beta \sum_{i=1}^{m}X_{i}^{2}+\frac{2r}{m+1}X_{m+1}^{2}-4\sum_{1Ci<j\leq m+1}X_{i}X_{j}.$$

By using $\sum_{i=1}^{m+1}X_{i}=0$ in (36), it follows that:

(37) $$V\left(X,X\right)=\beta \sum_{i=1}^{m}X_{i}^{2}+\frac{2r}{m+1}X_{m+1}^{2}+4\sum_{i=1}^{m+1}X_{i}^{2}\geq 0.$$

By virtue of the Remark 1, the critical point $\left(h_{11}^{0\alpha },...,h_{m+1\;m+1}^{0\alpha }\right)$ is the global minimum point of the problem. In particular, we have $f_{\alpha }\left(h_{11}^{0\alpha },...,h_{m+1\;m+1}^{0\alpha }\right)=0$. As a result, we obtain the inequality P≥0, namely represented by the inequalities (22) and (23), related to the infimum and supremum, respectively, over all tangent hyperplanes *L* of $T_{x}M$.

Finally, we pursue the equality cases of the inequalities (22) and (23). For this purpose, we reveal the critical points of P, i.e., the solutions of following equations system:

$$\left\{\begin{array}{c} \frac{\partial P}{\partial h_{ii}^{0\alpha }}=2\left[\frac{m+r+1}{m+1}+\frac{a(r)}{m}-1\right]h_{ii}^{0\alpha }-2\sum_{k\neq i,k=1}^{m+1}h_{kk}^{0\alpha }=0,\\ \frac{\partial P}{\partial h_{m+1\;m+1}^{0\alpha }}=\frac{2r}{m+1}h_{m+1\;m+1}^{0\alpha }-2\sum_{k=1}^{m}h_{kk}^{0\alpha }=0,\\ \frac{\partial P}{\partial h_{ij}^{0\alpha }}=4\left[\frac{m+r+1}{m+1}+\frac{a(r)}{m}\right]h_{ij}^{0\alpha }=0,\;\;i\neq j,\\ \frac{\partial P}{\partial h_{i\;m+1}^{0\alpha }}=\frac{4(m+r+1)}{m+1}h_{i\;m+1}^{0\alpha }=0. \end{array}\right.$$

Since $\bar{M}$ is a Kenmotsu statistical manifold, then we obtain the solution $h^{c}=h_{ij}^{0\alpha }=0$, for all i,j∈{1,...,m+1} and α∈{m+2,...,2n+1}. Moreover, due to P≥0 and $P(h^{c})=0$, then P has a minimum point $h^{c}$ indicated above. In conclusion, the case of equality of any of the inequalities (22) and (23) holds if and only if $h_{ij}^{\alpha }=-h_{ij}^{*\alpha },$ for i,j∈{1,...,m+1}, α∈{m+2,...,2n+1}. □

As a consequence of Theorem 2, we can derive the following inequalities involving the normalized δ-Casorati curvatures ${\hat{\delta }}_{C}\left(m\right)$ and $\delta_{C}\left(m\right)$, the dual normalized δ-Casorati curvatures $\delta_{C}^{*}\left(m\right)$ and ${\hat{\delta }}_{C}^{*}\left(m\right)$, as well as the normalized scalar curvature ρ of the submanifold.

**Theorem** **3.** *Let ($\bar{M},\bar{\nabla },g,\phi ,\xi$) be a (2n+1)-dimensional Kenmotsu statistical manifold of constant ϕ-sectional curvature c, endowed with a semi-symmetric metric connection $\tilde{\nabla }$. Suppose M is an (m+1)-dimensional statistical submanifold of ($\bar{M},\bar{\nabla },g,\phi ,\xi$) such that ξ is a tangent vector field on M. Then the normalized δ-Casorati curvatures fulfill the following inequalities:*

*(i)*  (38) $$\begin{array}{c} \delta_{C}^{0}\left(m\right)\geq \rho -\frac{3(c+1)}{4m(m+1)}{∥P∥}^{2}+\frac{c+1}{2(m+1)}-\frac{c-3}{4}+\frac{2}{m+1}\;trace\left(\gamma \right), \end{array}$$
  where $\delta_{C}^{0}\left(m\right)$ is defined by $2\delta_{C}^{0}\left(m\right)=\delta_{C}\left(m\right)+\delta_{C}^{*}\left(m\right)$, and
*(ii)*  (39) $$\begin{array}{c} {\hat{\delta }}_{C}^{0}\left(m\right)\geq \rho -\frac{3(c+1)}{4m(m+1)}{∥P∥}^{2}+\frac{c+1}{2(m+1)}-\frac{c-3}{4}+\frac{2}{m+1}\;trace\left(\gamma \right), \end{array}$$
  where ${\hat{\delta }}_{C}^{0}\left(m\right)$ is defined by $2{\hat{\delta }}_{C}^{0}\left(m\right)={\hat{\delta }}_{C}\left(m\right)+{\hat{\delta }}_{C}^{*}\left(m\right)$.

*Furthermore, the case of equality in any of the inequalities *(38)* and* (39)* holds at all points p∈M if and only if:*

$$\begin{array}{c} h=-h^{*}. \end{array}$$

**Proof.**  The inequality (38) follows replacing $r=\frac{m(m+1)}{2}$ in (22), by using (19) and remarking that we have the relation

$$\delta_{C}^{0}\left(m\left(m+1\right)/2;m\right)=m\left(m+1\right)\delta_{C}^{0}\left(m\right).$$

Similarly, we obtain inequality (39) replacing r=2m(m+1) in (23), by taking account of (19) and

$${\hat{\delta }}_{C}^{0}\left(2m\left(m+1\right);m\right)=m\left(m+1\right){\hat{\delta }}_{C}^{0}\left(m\right).$$

□

**Remark** **2.** *As proved in Theorems 2 and 3, the equality case of any of the inequalities* (22), (23), (38) *and* (39) is attained for those statistical submanifolds for which the imbedding curvature tensors h and $h^{*}$ are related by $h=-h^{*}$. Note that, in view of (12), *this condition implies the vanishing of the second fundamental form of the semi-symmetric metric connection. Hence, the equality case of any of the inequalities *(22), (23), (38) *and* (39) *holds at all points only for statistical submanifolds that are totally geodesic with respect to the semi-symmetric metric connection, or equivalently with respect to the Levi-Civita connection. This is a consequence of a result recently stated in* [39] *(see Corollary 4.4), where it was proved that for a statistical submanifold of a statistical manifold equipped with a semi-symmetric metric connection $\tilde{\nabla }$, the second fundamental form of the Levi-Civita connection coincides with the second fundamental form of $\tilde{\nabla }$.*

## 4. Example

Let us consider the (2n+1)-dimensional Kenmotsu statistical manifold $(H^{2n+1},$$\bar{\nabla }={\bar{\nabla }}^{0}+\bar{K},g,\phi ,\xi )$ constructed in [25] (for details see Examples 3.3 and 3.10 in the above referenced article). For the sake of simplicity, we will limit to the case of dimension 5, but the example we are going to build can be extended to any odd dimension. We remind that

$$H^{5}=\left\{\left(x_{1},x_{2},y_{1},y_{2},z\right)\in R^{5}|z>0\right\}$$

and the structure tensors (g,ϕ,ξ) are defined by

$$g=\frac{1}{z^{2}}\left\{{\left(dx_{1}\right)}^{2}+{\left(dx_{2}\right)}^{2}+{\left(dy_{1}\right)}^{2}+{\left(dy_{2}\right)}^{2}+{\left(dz\right)}^{2}\right\},$$

$$\phi \frac{\partial }{\partial x_{1}}=\frac{\partial }{\partial y_{1}},\;\phi \frac{\partial }{\partial x_{2}}=\frac{\partial }{\partial y_{2}},\;\phi \frac{\partial }{\partial y_{1}}=-\frac{\partial }{\partial x_{1}},\;\phi \frac{\partial }{\partial y_{2}}=-\frac{\partial }{\partial x_{2}},\;\phi \frac{\partial }{\partial z}=0$$

and

$$\xi =-z\frac{\partial }{\partial z}.$$

Denote by $\bar{\nabla }$ and ${\bar{\nabla }}^{*}$ the dual connections on $H^{5}$ such that $\bar{\nabla }={\bar{\nabla }}^{0}+\bar{K}$. We obtain:

$${\bar{\nabla }}_{\partial x_{1}}\partial x_{1}={\bar{\nabla }}_{\partial x_{2}}\partial x_{2}={\bar{\nabla }}_{\partial y_{1}}\partial y_{1}={\bar{\nabla }}_{\partial y_{2}}\partial y_{2}=0,$$

$${\bar{\nabla }}_{\partial x_{1}}\partial x_{2}={\bar{\nabla }}_{\partial x_{2}}\partial x_{1}={\bar{\nabla }}_{\partial y_{1}}\partial y_{2}={\bar{\nabla }}_{\partial y_{2}}\partial y_{1}=0,$$

$${\bar{\nabla }}_{\partial x_{1}}\partial y_{1}={\bar{\nabla }}_{\partial y_{1}}\partial x_{1}={\bar{\nabla }}_{\partial x_{2}}\partial y_{1}={\bar{\nabla }}_{\partial y_{1}}\partial x_{2}=0,$$

$${\bar{\nabla }}_{\partial x_{1}}\partial y_{2}={\bar{\nabla }}_{\partial y_{2}}\partial x_{1}={\bar{\nabla }}_{\partial x_{2}}\partial y_{2}={\bar{\nabla }}_{\partial y_{2}}\partial x_{2}=0,$$

$${\bar{\nabla }}_{\partial x_{1}}\partial z={\bar{\nabla }}_{\partial z}\partial x_{1}=-\frac{2}{z}\partial x_{1},\;{\bar{\nabla }}_{\partial x_{2}}\partial z={\bar{\nabla }}_{\partial z}\partial x_{2}=-\frac{2}{z}\partial x_{2},$$

$${\bar{\nabla }}_{\partial y_{1}}\partial z={\bar{\nabla }}_{\partial z}\partial y_{1}=-\frac{2}{z}\partial y_{1},\;{\bar{\nabla }}_{\partial y_{2}}\partial z={\bar{\nabla }}_{\partial z}\partial y_{2}=-\frac{2}{z}\partial y_{2},$$

$${\bar{\nabla }}_{\partial z}\partial z=-\frac{3}{z}\partial z.$$

Moreover, we obtain:

$${\bar{\nabla }}_{\partial x_{1}}^{*}\partial x_{1}={\bar{\nabla }}_{\partial x_{2}}^{*}\partial x_{2}={\bar{\nabla }}_{\partial y_{1}}^{*}\partial y_{1}={\bar{\nabla }}_{\partial y_{2}}^{*}\partial y_{2}=\frac{2}{z}\partial z,$$

$${\bar{\nabla }}_{\partial x_{1}}^{*}\partial x_{2}={\bar{\nabla }}_{\partial x_{2}}^{*}\partial x_{1}={\bar{\nabla }}_{\partial y_{1}}^{*}\partial y_{2}={\bar{\nabla }}_{\partial y_{2}}^{*}\partial y_{1}=0,$$

$${\bar{\nabla }}_{\partial x_{1}}^{*}\partial y_{1}={\bar{\nabla }}_{\partial y_{1}}^{*}\partial x_{1}={\bar{\nabla }}_{\partial x_{2}}^{*}\partial y_{1}={\bar{\nabla }}_{\partial y_{1}}^{*}\partial x_{2}=0,$$

$${\bar{\nabla^{*}}}_{\partial x_{1}}\partial y_{2}={\bar{\nabla }}_{\partial y_{2}}^{*}\partial x_{1}={\bar{\nabla^{*}}}_{\partial x_{2}}\partial y_{2}={\bar{\nabla }}_{\partial y_{2}}^{*}\partial x_{2}=0,$$

$${\bar{\nabla }}_{\partial x_{1}}^{*}\partial z={\bar{\nabla }}_{\partial z}^{*}\partial x_{1}={\bar{\nabla }}_{\partial x_{2}}^{*}\partial z={\bar{\nabla }}_{\partial z}^{*}\partial x_{2}=0,$$

$${\bar{\nabla }}_{\partial y_{1}}^{*}\partial z={\bar{\nabla }}_{\partial z}^{*}\partial y_{1}={\bar{\nabla^{*}}}_{\partial y_{2}}\partial z={\bar{\nabla }}_{\partial z}^{*}\partial y_{2}=0,$$

$${\bar{\nabla }}_{\partial z}^{*}\partial z=\frac{1}{z}\partial z.$$

For any $X,Y\in \Gamma (TH^{5})$, we assume that the (1,2)-tensor field $\bar{K}$ is given by:

$$\bar{K}\left(X,Y\right)=\nu \;\bar{\eta }\left(X\right)\;\bar{\eta }\left(Y\right)\;\xi ,$$

where $\nu \in C^{\infty }\left(H^{5}\right)$ and $\bar{\eta }$ is the 1-form on $H^{5}$ dual to ξ, that is $\bar{\eta }\left(X\right)=g\left(X,\xi \right)$.

Thus, it is known that $\left(H^{5},\bar{\nabla }={\bar{\nabla }}^{0}+\bar{K},g,\phi ,\xi \right)$ is a Kenmotsu statistical manifold with constant ϕ-sectional curvature c=−1 (see ([25]).

Next, we prove that $H^{5}$ admits a semi-symmetric metric connection. First, we assume that $\tilde{\nabla }$ is an affine connection defined as follows:

$${\tilde{\nabla }}_{\partial x_{1}}\partial x_{1}={\tilde{\nabla }}_{\partial x_{2}}\partial x_{2}={\tilde{\nabla }}_{\partial y_{1}}\partial y_{1}={\tilde{\nabla }}_{\partial y_{2}}\partial y_{2}=\frac{z-1}{z^{2}}\partial z,$$

$${\tilde{\nabla }}_{\partial x_{1}}\partial x_{2}={\tilde{\nabla }}_{\partial x_{2}}\partial x_{1}={\tilde{\nabla }}_{\partial y_{1}}\partial y_{2}={\tilde{\nabla }}_{\partial y_{2}}\partial y_{1}=0,$$

$${\tilde{\nabla }}_{\partial x_{1}}\partial y_{1}={\tilde{\nabla }}_{\partial y_{1}}\partial x_{1}={\tilde{\nabla }}_{\partial x_{2}}\partial y_{1}={\tilde{\nabla }}_{\partial y_{1}}\partial x_{2}=0,$$

$${\tilde{\nabla }}_{\partial x_{1}}\partial y_{2}={\tilde{\nabla }}_{\partial y_{2}}\partial x_{1}={\tilde{\nabla }}_{\partial x_{2}}\partial y_{2}={\tilde{\nabla }}_{\partial y_{2}}\partial x_{2}=0,$$

$${\tilde{\nabla }}_{\partial x_{1}}\partial z=\frac{1-z}{z^{2}}\partial x_{1},\;{\tilde{\nabla }}_{\partial x_{2}}\partial z=\frac{1-z}{z^{2}}\partial x_{2},$$

$${\tilde{\nabla }}_{\partial z}\partial x_{1}=-\frac{1}{z}\partial x_{1},\;{\tilde{\nabla }}_{z}\partial x_{2}=-\frac{1}{z}\partial x_{2},$$

$${\tilde{\nabla }}_{\partial y_{1}}\partial z=\frac{1-z}{z^{2}}\partial y_{1},\;{\tilde{\nabla }}_{\partial y_{2}}\partial z=\frac{1-z}{z^{2}}\partial y_{2},$$

$${\tilde{\nabla }}_{\partial z}\partial y_{1}=-\frac{1}{z}\partial y_{1},\;{\tilde{\nabla }}_{z}\partial y_{2}=-\frac{1}{z}\partial y_{2},$$

$${\tilde{\nabla }}_{\partial z}\partial z=-\frac{1}{z}\partial z.$$

Then the torsion tensor $\tilde{T}$ of $\tilde{\nabla }$ satisfies the relations:

$$\tilde{T}\left(\partial x_{i},\partial x_{j}\right)=\tilde{T}\left(\partial y_{i},\partial y_{j}\right)=\tilde{T}\left(\partial x_{i},\partial y_{j}\right)=0,$$

$$\tilde{T}\left(\partial x_{i},\partial z\right)=\frac{1}{z^{2}}\partial x_{i},\;\tilde{T}\left(\partial y_{i},\partial z\right)=\frac{1}{z^{2}}\partial y_{i},$$

for all i,j∈{1,2}.

It follows that $\tilde{\nabla }$ is a semi-symmetric connection satisfying (7) with $\eta =-\frac{1}{z}\bar{\eta }$. Furthermore, the relation $\tilde{\nabla }g=0$ holds, which implies that $\tilde{\nabla }$ is a semi-symmetric metric connection on the Kenmotsu statistical manifold $\left(H^{5},\bar{\nabla }={\bar{\nabla }}^{0}+\bar{K},g,\phi ,\xi \right)$ of constant ϕ-sectional curvature −1.

Let *M* be a 3-dimensional submanifold of the Kenmotsu statistical manifold $H^{5}$ with coordinates $\left(u_{1},u_{2},u_{3}\right)$ given by:

$$u:M\to H^{5},$$

$$u\left(u_{1},u_{2},u_{3}\right)=\left(\frac{1}{2}u_{1},\frac{1}{2}u_{2},-\frac{1}{2}u_{2},\frac{1}{2}u_{1},u_{3}\right).$$

Consider the following bases in the tangent bundle TM and normal bundle $T^{⊥}M$, respectively:

$$\left\{T_{1}=\frac{1}{2}\left(\partial x_{1}+\partial y_{2}\right),T_{2}=\frac{1}{2}\left(\partial x_{2}-\partial y_{1}\right),T_{3}=\partial z\right\}$$

and

$$\{N_{1}=\frac{1}{2}\left(\partial x_{1}-\partial y_{2}\right),N_{2}=\frac{1}{2}\left(\partial x_{2}+\partial y_{1}\right)\}.$$

Then we obtain:

$${\tilde{\nabla }}_{T_{1}}T_{1}=\frac{z-1}{2z^{2}}T_{3},\;{\tilde{\nabla }}_{T_{2}}T_{2}=0,\;{\tilde{\nabla }}_{T_{3}}T_{3}=-\frac{1}{z}T_{3},$$

$${\tilde{\nabla }}_{T_{1}}T_{2}={\tilde{\nabla }}_{T_{2}}T_{1}=0,$$

$${\tilde{\nabla }}_{T_{1}}T_{3}=\frac{1-z}{z^{2}}T_{1},\;{\tilde{\nabla }}_{T_{3}}T_{1}=-\frac{1}{z}T_{1},$$

$${\tilde{\nabla }}_{T_{2}}T_{3}=\frac{1-z}{z^{2}}T_{2},\;{\tilde{\nabla }}_{T_{3}}T_{2}=-\frac{1}{z}T_{2}$$

and it follows immediately that the submanifold *M* is totally geodesic with respect to the semi-symmetric metric connection $\tilde{\nabla }$. Moreover, we conclude that the inequalities (22), (23), (38) and (39) are all satisfied with equality sign.

## 5. Conclusions

The purpose of this paper is to establish new inequalities between intrinsic and extrinsic curvature invariants, related to the normalized δ-Casorati curvatures and the scalar curvature of statistical submanifolds in Kenmotsu statistical manifolds of constant ϕ-sectional curvature, which are endowed with semi-symmetric metric connection. In addition, we pursued the equality cases of these inequalities and provided a nontrivial example to illustrate the results. Therefore, we believe that the topic of this survey may be developed in new challenging approaches on various classes of submanifolds in some statistical manifolds endowed with semi-symmetric metric connection.

## References

[1] Chen B.-Y. (1999). Relations between Ricci curvature and shape operator for submanifolds with arbitrary codimension. Glasgow Math. J..

[2] Chen B.-Y. (2011). Pseudo-Riemannian Geometry, δ-Invariants and Applications.

[3] Chen B.-Y. (2021). Recent developments in *δ*-Casorati curvature invariants. Turk. J. Math..

[4] Chen B.-Y. (2021). Recent developments in Wintgen inequality and Wintgen ideal submanifolds. Int. Electron. J. Geom..

[5] Chen B.-Y., Decu S., Vîlcu G.-E. (2021). Inequalities for the Casorati Curvature of Totally Real Spacelike Submanifolds in Statistical Manifolds of Type Para-Kähler Space Forms. Entropy.

[6] Chen B.-Y., Blaga A.M., Vîlcu G.-E. (2022). Differential Geometry of Submanifolds in Complex Space Forms Involving *δ*-Invariants. Mathematics.

[7] Mihai I., Van der Veken J., Carriazo A., Dimitrić I., Oh Y.M., Suceavă B., Vrancken L. (2020). Statistical manifolds and their submanifolds. Results on Chen-like invariants. Geometry of Submanifolds: AMS Special Session in Honor of Bang-Yen Chen’s 75th Birthday, 20–21 October 2018, University of Michigan, Ann Arbor, Michigan.

[8] Decu S., Haesen S., Verstraelen L., Vîlcu G.-E. (2018). Curvature Invariants of Statistical Submanifolds in Kenmotsu Statistical Manifolds of Constant *ϕ*-Sectional Curvature. Entropy.

[9] Decu S., Haesen S., Verstraelen L. (2020). Inequalities for the Casorati Curvature of Statistical Manifolds in Holomorphic Statistical Manifolds of Constant Holomorphic Curvature. Mathematics.

[10] Decu S., Haesen S. (2022). Chen Inequalities for Spacelike Submanifolds in Statistical Manifolds of Type Para-Kähler Space Forms. Mathematics.

[11] Chen B.-Y. (1993). Some pinching and classification theorems for minimal submanifolds. Arch. Math..

[12] Decu S., Haesen S., Verstraelen L. (2007). Optimal inequalities involving Casorati curvatures. Bull. Transilv. Univ. Braşov Ser. B Suppl..

[13] Decu S., Haesen S., Verstraelen L. (2008). Optimal inequalities characterising quasi-umbilical submanifolds. J. Inequal. Pure Appl. Math..

[14] Casorati F. (1890). Mesure de la courbure des surfaces suivant l’idée commune. Acta Math..

[15] Verstraelen L. (2013). Geometry of submanifolds I. The first Casorati curvature indicatrices. Kragujevac J. Math..

[16] Koenderink J.J. (2012). Shadows of Shapes.

[17] Koenderink J., van Doorn A., Wagemans J. (2015). Local solid shape. i-Perception.

[18] Amari S., Berger J., Fienberg S., Gani J., Krickeberg K., Olkin I., Singer B. (1985). Differential-Geometrical Methods in Statistics. Lecture Notes in Statistics.

[19] Vîlcu G.-E. (2021). Almost products structures on statistical manifolds and para-Kähler-like statistical submersions. Bull. Sci. Math..

[20] Gomez I.S., Portesi M., Borges E.P. (2020). Universality classes for the Fisher metric derived from relative group entropy. Phys. A Stat. Mech. Appl..

[21] Jäntschi L., Balint D., Bolboacă S.D. (2016). Multiple linear regressions by maximizing the likelihood under assumption of generalized Gauss-Laplace distribution of the error. Comput. Math. Methods Med..

[22] Pessoa P., Costa F.X., Caticha A. (2021). Entropic Dynamics on Gibbs Statistical Manifolds. Entropy.

[23] Kenmotsu K. (1972). A class of almost contact Riemannian manifolds. Tohoku Math. J..

[24] Pitiş G., Haesen S., Verstraelen L. (2017). Contact Forms in Geometry and Topology. Topics in Modern Differential Geometry.

[25] Furuhata H., Hasegawa I., Okuyama Y., Sato K. (2017). Kenmotsu statistical manifolds and warped product. J. Geom..

[26] Hayden H.A. (1932). Subspaces of a space with torsion. Proc. London Math. Soc..

[27] Yano K. (1970). On semi-symmetric metric connection. Rev. Roumaine Math. Pures Appl..

[28] Imai T. (1972). Notes on semi-symmetric metric connections. Tensor.

[29] Imai T. (1972). Hypersurfaces of a Riemannian manifold with semi-symmetric metric connection. Tensor.

[30] Nakao Z. (1976). Submanifolds of a Riemannian manifold with semisymmetric metric connections. Proc. Amer. Math. Soc..

[31] Decu S. (2009). Optimal inequalities for submanifolds in quaternion-space-forms with semi-symmetric metric connection. Bull. Transilv. Univ. Braşov.

[32] He G., Liu H., Zhang L. (2016). Optimal inequalities for the Casorati curvatures of submanifolds in generalized space forms endowed with semi-symmetric non-metric connections. Symmetry.

[33] Lee C.W., Lee J.W., Vîlcu G.-E., Yoon D.W. (2015). Optimal inequalities for the Casorati curvatures of submanifolds of generalized space forms endowed with semi-symmetric metric connections. Bull. Korean Math. Soc..

[34] Mihai A., Özgür C. (2010). Chen inequalities for submanifolds of real space forms with a semi-symmetric metric connection. Taiwan. J. Math..

[35] Poyraz N., Dogan B., Yaşar E. (2017). Chen inequalities on lightlike hypersurface of a Lorentzian manifold with semi-symmetric metric connection. Int. Electron. J. Geom..

[36] Zhang P., Zhang L. (2016). Casorati Inequalities for Submanifolds in a Riemannian Manifold of Quasi-Constant Curvature with a Semi-Symmetric Metric Connection. Symmetry.

[37] Zhang P., Zhang L., Song W. (2014). Chen’s inequalities for submanifolds of a Riemannian manifold of quasi-constant curvature with a semi-symmetric metric connection. Taiwan J. Math..

[38] Kazan S., Kazan A. (2018). Sasakian Statistical Manifolds with Semi-Symmetric Metric Connection. Univers. J. Math. Appl..

[39] Balgeshir M.B.K., Salahvarzi S. (2021). Curvatures of semi-symmetric metric connections of statistical manifolds. Commun. Korean Math. Soc..

[40] Furuhata H., Hasegawa I., Dragomir S., Shahid M.H., Al-Solamy F.R. (2016). Submanifold theory in holomorphic statistical manifolds. Geometry of Cauchy-Riemann Submanifolds.

[41] Vos P. (1989). Fundamental equations for statistical submanifolds with applications to the Barlett correction. Ann. Inst. Statist. Math..

[42] Oprea T. (2006). Constrained Extremum Problems in Riemannian Geometry.

[43] Vîlcu G.-E. (2018). An optimal inequality for Lagrangian submanifolds in complex space forms involving Casorati curvature. J. Math. Anal. Appl..

